# Quo vadis biodiversity? Species richness following twenty years of taxonomic revisions on Afrotropical Galerucinae *s. str.* (Coleoptera, Chrysomelidae)

**DOI:** 10.3897/zookeys.720.14011

**Published:** 2017-12-11

**Authors:** Thomas Wagner

**Affiliations:** 1 Universität Koblenz-Landau, Institut für Integrierte Naturwissenschaften – Biologie, Universitätsstr. 1, D-56070 Koblenz, Germany

**Keywords:** Africa, Afrotropical, region, biodiversity, Galerucinae s. str., Monoleptites, revision, taxonomy

## Abstract

Galerucinae is one of the most species-rich leaf beetle group with its greatest diversity occurring in tropical forests. There are 1680 nominal species of Afrotropical Galerucinae s. str. (without Alticini) described. Considering global diversity estimations, many unknown species can be presumed. Several taxa traditionally placed in “Monoleptites”, have been revised in the last twenty years. To date 259 species have been re-examined, revealing in 139 valid species and 120 mainly newly recognized synonyms. This large number of synonyms can mainly be ascribed to highly variable colour patterns, a typical character used for the identification of many chrysomelid species. Genitalic structures and molecular work can support a more precise allocation to species. Within around 72,000 specimens of galerucines s. str. from 48 museums and private collections, only 107 species were newly described. After revising approximately 15% of the Afrotropical galerucine fauna their species richness decreased from 259 to 246 species, a pattern that appears to be similar to that for other African galerucine groups. Since the estimation of the extent of global diversity based mainly on insect species richness in tropical forests, our current study which is based on hard data suggests a much lower diversity than previously predicted.

## Introduction

Galerucinae s. str. (without Alticini) is one of most diverse group of leaf beetles in tropical forests, including 1680 nominal species from Africa (Wagner 2006) and 7145 species worldwide (Nie et al. 2017). Among the highly diverse Galerucinae, *Monolepta* Chevrolat, 1836 is the largest genus of Galerucinae s. str., with nearly 700 described species in the world (Wagner 2007a). When a taxonomic and phylogenetic revision of Afrotropical *Monolepta* was started, it became clear that this genus as traditionally delimited was a non-monophyletic group (Wagner 1999, 2003, 2004). *Monolepta* and other taxa with a distinctly elongated first tarsomere of the hind-leg are placed in “Monoleptites” (Wilcox 1973). Subsequently, the relative length of the second to third antennomeres, and the shape of the pronotum were considered to place the “long-legged” African galerucines largely in three genera: *Monolepta* (second and third antennomere of same length, pronotum rectangular); *Candezea* Chapuis, 1879 (third antennomere much longer than second, pronotum rectangular); and *Barombiella* Laboissière, 1931 (third antennomere much longer than second, pronotum trapezoidal).

During a period of twenty years, our working group has revised approximately 85 % of the “Monoleptites” sensu Wilcox (1973). Besides redefining generic characters in the external morphology, we also studied the genitalic patterns of all the examined taxa for the first time. These were found to be valuable not only to distinguish species, but also to define genera as monophyletic groups within *Monolepta* (e. g. Wagner 2007a), *Candezea* (Wagner and Kurtscheid 2005), and *Barombiella* (Freund and Wagner 2003, Wagner and Freund 2003, Bolz and Wagner 2011). Some species were transferred to *Afrocrania* Hincks, 1949 (Middelhauve and Wagner 2001, Wagner 2007b). We found several phylogenetically isolated taxa that had to be transferred to newly described genera, e. g. *Afromaculepta* (Hasenkamp and Wagner 2000), *Afrocandezea* (Wagner and Scherz 2002, Scherz and Wagner 2007), *Afronaumannia* (Steiner and Wagner 2005), *Monoleptoides* (Wagner 2011), and *Bicolorizea* (Heunemann et al. 2015). These supra-specific taxa could be also identified as separate phylogenetic units by molecular data (Wagner in prep.). We included also short-legged *Bonesioides* Laboissière, 1925 (Freund and Wagner 2003), *Galerudolphia* Hincks, 1949 (Bolz and Wagner 2005) and the very short-legged *Ootheca* Chevrolat, 1836 in our revisions (Kortenhaus and Wagner 2010, 2011, 2012, 2013).

At present, some 250 species of Afrotropical Galerucinae *s. str.* have been revised and these data are used here as a case study on their general species richness. Global insect diversity caught the attention of entomologists, and a broader audience, in the 1980s, when data of canopy fogging in tropical forests were extrapolated to 30 million species of insects (Erwin 1982). This started a controversial discussion in the community (e.g., Stork 1988, Thomas 1990, Gaston 1991), but more detailed “calculations” led to a much lower number that levelled off at around six million species (Ødegaard 2000, Basset et al. 2012). The author’s empirical data of species revisions in a quite highly diverse tropical leaf-beetle group is used here to address the question, What is the global diversity of Galerucinae s. str.?

## Material and methods

Our revisions of Afrotropical galerucines are currently published in 40 papers with a taxonomic focus (Wagner and collaborators 1993–2017) based on around 72,000 specimens from 48 collections which includes all the major museum collections that house African insects.

## Results

**Figure 1.**
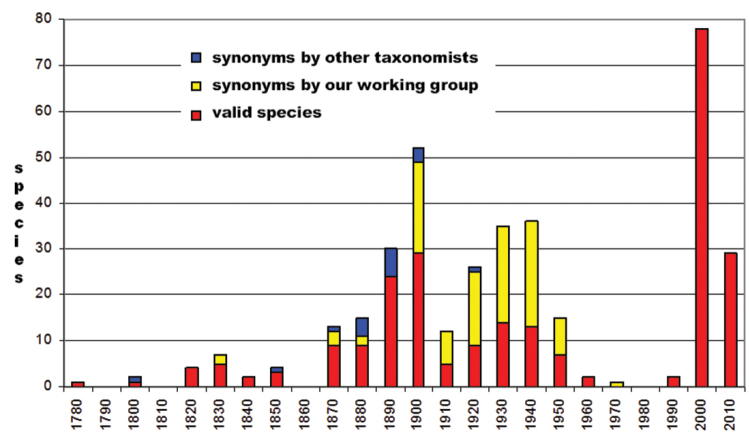
Numbers of described species of Afrotropical Galerucinae s. str. per decade revised between 2000 to 2016 by our working group with synonyms found.

Up to now, 259 species have been re-examined, resulting in 139 valid species and 120, mainly newly recognized synonyms (Fig. 1). The high variability of colour pattern, a typical character for many chrysomelid species, caused the high number of synonyms (46%). Genitalic structures and molecular data can make more reliable species identification. The large number of specimens examined revealed only 107 new species described. After revising approximately 15% of the Afrotropical galerucine fauna, the species number decreased from 259 to 246 species.

## Discussion

The high polychromatism in many galerucine species is the cause of the majority of synonyms, since colour patterns were used by previous authors as very definite species specific characters. When species are widely distributed, the number of synonyms increase even more. *Monolepta vincta* Gerstaecker, 1871, has a pan-Afrotropical distribution and ten synonyms have been found for his species (Wagner 2005), six of these synonyms are provided in two publications of Victor Laboissière (1920a, b). This is not a reproach for Laboissière, who was the most productive taxonomist on the Galerucinae world-wide. The majority of species described by him are still valid, but in his early publications, his work was based on a very “classic”, Linnaean species concept, as it was customary for that time. Later in his career (e.g. Laboissière 1940), he used genitalic patterns for species identification, being one of the first taxonomists in Chrysomelidae to do so. Further examples of widely distributed African galerucines with a large number of synomyns are *Neobarombiella flavilabris* (Weise, 1903) with eleven and *Neobarombiella senegalensis* (Laboissière, 1923) with ten synonyms.

Other diverse Afrotropical Galerucinae s. str. appear to indicate a similar “over-description” rate. *Diacantha* Chevrolat, 1836 (syn. *Hyperacantha* Chapuis, 1879) is the second most diverse group of African galerucines in terms of some 120 described species. A few spot checks revealed there are a large number of synonyms in this genus too, and *Diacantha* might be another taxonomic “nightmare”, revealing a lower number of valid species subsequent to formal revision.

On the other hand, revisions of tropical phytophagous insects can result in a strong increased number of species. Examples are the weevil genera *Euops* Schönherr, 1839 from New Guinea with 24 valid species before revision, and 160 additional new species there after (Riedel 2006), and the litter-dwelling *Trigonopterus* Fauvel, 1862 from the Sundaland area (mainly Malaysia, Indonesia) which was monotypic and comprised 98 species after being revised (Riedel et al. 2014). Alexander Riedel’s studies on East Asian weevils revealed six times more species after the revision of *Euops*, and a much larger increase in *Trigonopterus* with their cryptic life-history, whereas our conclusion brought decreased species richness to light. The results may reflect the two extremes along a continuum, but both data sets imply that more recent “calculations” on global insect diversity, with around six million species, are much more reasonable than the higher numbers “believed” before.

## References

[B1] Basset Y et al. (2012) Arthropod diversity in a tropical forest. Science 338: 1481–1484. https://doi.org/10.1126/science.1226727 23239740 10.1126/science.1226727

[B2] Bolz H Wagner T (2005) Revision of *Galerudolphia* from tropical Africa (Coleoptera, Chrysomelidae, Galerucinae). Insect Systematics and Evolution 35: 361–400. https://doi.org/10.1163/187631204788912436

[B3] Bolz H Wagner T (2012) *Neobarombiella*, a diverse, newly described genus of Afrotropical Galerucinae (Coleoptera, Chrysomelidae). Zootaxa 3463: 1–112.

[B4] Erwin TL (1982) Tropical forests: their richness in Coleoptera and other arthropod species. The Coleopterists Bulletin 36: 74–75.

[B5] Freund W Wagner T (2003) Revision of *Bonesioides* Laboissière, 1925 (Coleoptera; Chrysomelidae; Galerucinae) from continental Africa. Journal of Natural History 37: 1915–1976. https://doi.org/10.1080/00222930110096519

[B6] Gaston KJ (1991) The magnitude of global insect species richness. Conservation Biology 5: 283–296. https://doi.org/10.1111/j.1523-1739.1991.tb00140.x

[B7] Hasenkamp R Wagner T (2000) Revision of *Afromaculepta* gen. n., a monophyletic group of Afrotropical galerucinae leaf beetles (Coleoptera: Chrysomelidae). Insect Systematics and Evolution 31: 3–26. https://doi.org/10.1163/187631200X00282

[B8] Heunemann LO Dalstein V Schulze M Wagner T (2015) *Bicolorizea* gen. nov. from tropical Africa (Coleoptera: Chrysomelidae, Galerucinae). Entomologische Zeitschrift 125(4): 235–246.

[B9] Kortenhaus S Wagner T (2010) Revision of *Ootheca* Chevrolat, 1837 from tropical Africa – Redescriptions, descriptions of new species and identification key (Coleoptera: Chrysomelidae: Galerucinae). Zootaxa 2659: 1–52.

[B10] Kortenhaus S Wagner T (2011) *Oothecoides* gen. nov. from tropical Africa, with redescriptions and description of six species (Coleoptera: Chrysomelidae: Galerucinae). Entomologische Zeitschrift 121: 259–269.

[B11] Kortenhaus S Wagner T (2012) Description of *Ootibia* gen. n. from tropical Africa with revision of two described species and description of three new species (Coleoptera: Chrysomelidae: Galerucinae). African Entomology 20(2): 350–370. https://doi.org/10.4001/003.020.0210

[B12] Kortenhaus S Wagner T (2013) *Oosagitta* gen. nov. from tropical Africa, with revision of two species and description of four new species (Coleoptera: Chrysomelidae, Galerucinae). European Journal of Taxonomy 58: 1–24. http://dx.doi.org/10.5852/ejt.2013.58

[B13] Laboissière V (1920a) Diagnoses de Galerucini nouveaux d’Afrique (Col. Chrysomelidae). Bulletin de la Société entomologique de France 1920: 50–53.

[B14] Laboissière V (1920b) Diagnoses de Galerucini nouveaux d’Afrique (Col. Chrysomelidae). Bulletin de la Société entomologique de France 1920: 98–101.

[B15] Laboissière V (1940) Galerucinae. Institut des Parcs Nationaux du Congo Belge, Bruxelles. Exploration du Parc National Albert 31: 1–98.

[B16] Middelhauve J Wagner T (2001) Revision of *Afrocrania* (Coleoptera: Chrysomelidae, Galerucinae). Part I: Species in which the males have head cavities or extended elytral extrusions. European Journal of Entomology 98: 511–531. https://doi.org/10.14411/eje.2001.066

[B17] Nie R-E Bezděk J Yang X-K (2017) How many genera and species of Galerucinae *s. str.* do we know? Updated statistics (Coleoptera, Chrysomelidae). In: Schmitt M Chaboo CS (Eds) Research on Chrysomelidae 7. ZooKeys 720: 91–102. https://doi.org/10.3897/zookeys.720.13517 10.3897/zookeys.720.13517 PMC5740445 29290727

[B18] Ødegaard F (2000) How many species of arthropods? Erwin’s estimate revised. Biological Journal of the Linnean Society 71: 583–597. https://doi.org/10.1111/j.1095-8312.2000.tb01279.x

[B19] Riedel A (2006) Revision of the subgenus Metaeuops Legalov of *Euops* Schoenherr (Coleoptera, Curculionoidea, Attelabidae) from the Papuan region. Zootaxa 1181: 1–102.

[B20] Riedel A Tänzler R Balke M Rahmadi C Suhardjono YR (2014) Ninety-eight new species of *Trigonopterus* weevils from Sundaland and the Lesser Sunda Islands. ZooKeys 467: 1–162. https://doi.org/10.3897/zookeys.467.8206 10.3897/zookeys.467.8206 PMC4296478 25610340

[B21] Scherz X Wagner T (2007) Revision of *Afrocandezea* Wagner & Scherz, 2002 from tropical Africa (Coleoptera: Chrysomelidae, Galerucinae). Entomologische Zeitschrift 117: 161–183.

[B22] Steiner I Wagner T (2005) *Afronaumannia* gen. nov., a new monophyletic group of leaf beetles from Africa (Coleoptera: Chrysomelidae, Galerucinae). Entomologische Zeitschrift 115: 15–24.

[B23] Stork NE (1988) Insect diversity: facts, fiction and speculation. Biological Journal of the Linnean Society 35: 321–337. https://doi.org/10.1111/j.1095-8312.1988.tb00474.x

[B24] Thomas CD (1990) Fewer species. Nature 347: 237. https://doi.org/10.1038/347237a0

[B25] Wagner T (1999) An introduction to the revision of afrotropical *Monolepta* and related taxa (Chrysomelidae, Coleoptera). In: Waloßek D (Ed.) Systematik im Aufbruch. Tagungsband zur ersten Jahrestagung der Gesellschaft für Biologische Systematik in Bonn vom 17.–19. September 1998, Courier Forschungsinstitut Senckenberg 215, Frankfurt, 215–220.

[B26] Wagner T (2003) Present status of a taxonomic revision of afrotropical *Monolepta* and related groups (Galerucinae). In: Furth DG (Ed.) Special Topics in Leaf Beetle Biology. Proceedings V International Symposium on the Chrysomelidae, Foz do Iguacu 2000. Pensoft, Sofia, 133–146.

[B27] Wagner T (2004) Phylogeny of Afrotropical *Monolepta* and related taxa (Galerucinae). In: Jolivet P, Santiago-Blay JA Schmitt M (Eds) New Developments in the Biology of Chrysomelidae, Academic Publishing, The Hague, 75–84.

[B28] Wagner T (2005) Revision of the vincta Species-group of Monolepta Chevrolat, 1837 from Africa, Arabia and the near East (Coleoptera, Chrysomelidae, Galerucinae). Bonner zoologische Beiträge 53: 255–282.

[B29] Wagner T (2006) AfriGa – An illustrated electronic catalogue of Afrotropical Galerucinae (Chrysomelidae, Coleoptera). Chrysomela 47: 7–8.

[B30] Wagner T (2007a) *Monolepta* Chevrolat, 1837, the most speciose galerucine taxon: redescription of the type species *Monolepta bioculata* (Fabricius, 1781) and key to related genera from (Chrysomelidae, Coleoptera). Journal of Natural History 41: 81–100. https://doi.org/10.1080/00222930601127384

[B31] Wagner T (2007b) Revision of *Afrocrania* (Coleoptera: Chrysomelidae: Galerucinae) Part II: Species in which the males lack head cavities or extended elytral extrusions. European Journal of Entomology 104: 801–814. https://doi.org/10.14411/eje.2007.101

[B32] Wagner T (2011) Description of *Monoleptoides* gen. nov. from the Afrotropical Region, including the revision of nine species (Coleoptera: Chrysomelidae, Galerucinae). Bonn Zoological Bulletin 66(2): 169–199.

[B33] Wagner T Freund W (2003) Revision of *Barombiella violacea* (Jacoby, 1984) (Coleoptera: Chrysomelidae, Galerucinae). Entomologische Zeitschrift 113: 258–262.

[B34] Wagner T Kurtscheid A (2005) Revision of *Candezea* from continental Africa (Coleoptera, Chrysomelidae, Galerucinae). Journal of Natural History 39: 2591–2641. https://doi.org/10.1080/00222930500102611

[B35] Wagner T Scherz X (2002) *Afrocandezea* gen. nov. from tropical Africa (Coleoptera: Chrysomelidae, Galerucinae). Entomologische Zeitschrift 112: 357–362.

[B36] Wilcox JA (1973) Chrysomelidae: Galerucinae: Luperini: Luperina. In: Junk W (Ed.) Coleopterorum Catalogus Suppl. 78: 433−664.

